# Efficacy and Safety of High-Dose Intravitreal Aflibercept in Neovascular Age-Related Macular Degeneration (nAMD): A Systematic Review

**DOI:** 10.7759/cureus.77906

**Published:** 2025-01-24

**Authors:** William Evans, Molly Evans, Mahmoud Eissa

**Affiliations:** 1 Medical School, University of Chester, Chester, GBR; 2 Department of Ophthalmology, Salisbury District Hospital, Salisbury, GBR; 3 Department of Ophthalmology, Mountainhall Treatment Centre, Dumfries, GBR

**Keywords:** anti-vegf treatment, a systematic review, best corrected visual acuity, central retinal thickness, efficacy of aflibercept, high dose aflibercept, intravitreal injection

## Abstract

Age-related macular degeneration (AMD) is one of the leading causes of blindness worldwide, and so continued research into new treatment options remains a priority. No systematic review has yet analysed the effectiveness and safety of higher-dose aflibercept across the available literature. We aimed to systematically evaluate the efficacy and safety of higher-dose intravitreal aflibercept (>2 mg) on Central Retinal Thickness (CRT) and Best Corrected Visual Acuity (BCVA) in patients with neovascular AMD (nAMD). In order to achieve this, a systematic literature search was conducted in January 2025 using PubMed and Ovid MEDLINE databases. Results were screened, and inclusion criteria were applied. Studies were included if they reported outcome data for intravitreal aflibercept doses above 2 mg for the treatment of nAMD. Of 382 identified articles, eight studies met inclusion criteria, encompassing data from 903 eyes. Analysis of all studies combined revealed a statistically significant improvement at 10 months, with a 33% reduction in CRT and a 29% improvement in BCVA. Subgroup analysis suggested greater effectiveness of 8 mg compared to 3 mg doses, particularly for CRT reduction (36% at 10 months vs. 28% at 12 months). However, small sample sizes and high heterogeneity across studies, including variations in dose, injection intervals, and patient characteristics (e.g., treatment-naïve vs. treatment-resistant), limit the reliability and conclusions of these outcome findings. There was a complication rate of 1.7% for serious adverse effects. In conclusion, higher doses of aflibercept (>2 mg) are safe and effective for treating nAMD, but further research is needed to analyse the comparison of different high doses and the effect of injection frequency on outcomes.

## Introduction and background

Age-related macular degeneration (AMD) is one of the leading causes of irreversible blindness worldwide, affecting an estimated 200 million people globally, typically those over 65 years of age [1,2].

Neovascular or "wet" AMD represents a severe form of the disease, characterised by the growth of abnormal blood vessels in the choroid. This leads to complications, such as fluid leakage into the retina and haemorrhages in the macula. Without treatment, this can cause permanent damage to the retinal photoreceptors and the retinal pigment epithelium, resulting in diminished eyesight and potentially permanent loss of vision [3,4].

The prevalence of AMD is expected to rise significantly in the upcoming years, increasing the burden on healthcare systems and substantially affecting the quality of life of affected patients. Therefore, continued research into effective treatment options remains crucial in addressing the growing demand [5,6].

The mainstay of current treatment for neovascular AMD (nAMD) primarily targets vascular endothelial growth factor (VEGF), a protein that plays a central role in angiogenesis and subsequent vascular leakage. The discovery of VEGF’s involvement in this condition has led to the development of anti-VEGF therapies, which have significantly improved the management of nAMD and patient outcomes, providing the first opportunity to restore vision [7,8]. It is estimated that anti-VEGF therapy has already reduced the risk of blindness due to nAMD by approximately 50% [8,9].

One such commonly used anti-VEGF agent is aflibercept (Eyelea), licensed in Europe in 2012 and approved by NICE (National Institute for Health and Care Excellence) in 2013 for the treatment of nAMD. Aflibercept is a fully human recombinant fusion protein, which inhibits VEGF-A, VEGF-B, and placental growth factor [7,8,10].

Initially administered as 2 mg, the efficacy of aflibercept in treating nAMD, particularly in improving Best Corrected Visual Acuity (BCVA) and reducing Central Retinal Thickness (CRT), has been extensively proven in multiple studies. These have shown it to be either non-inferior or potentially superior to other anti-VEGF agents, as well as allowing for an extended dosing interval while maintaining a similar safety profile [11-14]. Furthermore, aflibercept has proven effective for other retinal conditions, such as diabetic retinopathy and retinal vein occlusion, where, once again, it has a similar efficacy and safety profile to alternative therapies, especially in treatment-resistant patients [14-16].

The success of aflibercept has led to the exploration of higher dosages, namely 4 mg and 8 mg. Initial studies demonstrated an improvement in anatomical outcomes with the 4 mg dose, and subsequent simulation studies have suggested that increasing the dose to 8 mg may prolong VEGF inhibition [17-18]. In recent years, there has been ongoing research into the safety and anatomical outcomes of the 8 mg dose. Namely, positive findings have been reported from the PHOTON trial for diabetic macular oedema, as well as the CANDELA and PULSAR trials for nAMD [19-21]. Additionally, ongoing studies are exploring the potential of 8 mg aflibercept in other retinal conditions, such as the phase 3 QUASAR trial [22]. These results contributed to the approval of the 8 mg dose by the U.S. Food and Drug Administration (FDA) in August of 2023 [23].

Despite these promising results, it remains crucial to monitor both the efficacy and safety of high-dose aflibercept to evaluate any potential risks, such as intraocular inflammation, as reported in a recent case series [24]. Furthermore, as with any new treatment, it is important to consolidate all available evidence to fully evaluate its effectiveness and safety. To the authors’ knowledge, no systematic review has yet examined the efficacy of high-dose aflibercept.

This review, therefore, aims to evaluate the efficacy of high-dose aflibercept (above 2 mg) in nAMD, specifically in terms of CRT and BCVA, whilst also commenting on the safety profile across the current data.

## Review

Methods

This systematic review was conducted following the standards set by the Preferred Reporting Items for Systematic Reviews and Meta-analysis (PRISMA) guidelines, for systematic reviews.

Literature Search

A comprehensive electronic literature search was completed on the 4th of January 2025, across the PubMed and OVID Medline databases. Boolean operators were used to ensure specificity in the search results. The full search strategy is as follows: (Aflibercept OR Eyelea OR Zaltrap) AND (High dose OR 4mg OR 8mg) AND (Intravitreal OR nAMD OR AMD OR age-related macular degeneration OR neovascular) AND (BCVA OR CST OR CRT OR Efficacy OR effectiveness OR outcome OR safety OR adverse effect OR complication* OR side effect* OR toxicity OR tolerability).

Filters for human subjects and the English language were applied automatically, and duplicate records were removed during the process. The search was designed to address the following research question: what is the effect of high-dose aflibercept on CRT and BCVA in nAMD?

Selection Criteria

The inclusion and exclusion criteria for this review were formulated using the PICOS (Population, Intervention, Comparator, Outcomes, and Study Design) framework.

The population of interest consisted of adults 18 years or older with nAMD. The intervention was high-dose aflibercept (dose greater than 2 mg). As our primary objective was to evaluate the efficacy of high-dose aflibercept, there was no specific comparator, since several systematic reviews have already established the efficacy of the 2 mg dose for this condition [13,25]. The outcomes were any reported changes in CRT and BCVA following intravitreal injections. We included cohort studies, randomised controlled trials (RCTs), and case series.

Studies were excluded if they involved doses of aflibercept less than or equal to 2 mg, if the indication was not nAMD, or if they did not clearly report changes in CRT or BCVA for individual higher dosages. Additionally, we excluded case reports, systematic reviews, conference abstracts, studies not involving adult human subjects, non-English language publications, or studies without full-text availability.

All articles identified through the search were initially screened based on their titles and abstracts. Full-text articles were then retrieved and further screened according to the inclusion criteria. This process was conducted manually by two independent reviewers (WE and ME). Disagreements were resolved through discussion with a third independent reviewer (ME) until an agreement was met. This method allowed for minimal selection bias throughout this process.

Data Extraction and Analysis

Data from the included studies was extracted and tabulated using Microsoft Excel (Microsoft® Corp., Redmond, WA, USA) by a single reviewer (WE), with quality checks performed by two additional reviewers (ME and ME). Captured data included study type, year of publication, aflibercept dose, frequency of injections, baseline BCVA and CRT, as well as values for BCVA and CRT at 1, 3, 6, 10, and 12 months where available. Any adverse effects were also recorded.

BCVA values were standardised into logMAR for analysis. For studies that reported BCVA in Early Treatment Diabetic Retinopathy Study (ETDRS) letters, values were converted to logMAR using the following formula [26]: \[-0.02 \times \text{ETDRS} + 1.7\]

In cases where data from the study was presented as graphs, quantitative values were extracted using an online digitisation tool. This allowed for the accurate conversion of the points on the graph into quantitative values, based on the axes provided.

If studies did not comment on participant completion rates or dropout rates, it was assumed that all eyes were analysed at each time point. In cases where participant numbers were only reported at certain points (e.g., baseline and conclusion), the lower number of participants was assumed for analysis.

Outcomes of Interest

The primary outcomes of interest were the aflibercept dose, changes in CRT, changes in BCVA, and the frequency of injections.

Quality Assessment

The quality of the studies was assessed by a single reviewer (WE), with quality assurance from two further reviewers (ME and ME). The risk of bias for RCTs was evaluated using the Cochrane Risk of Bias 2 tool [27]. For case series, the JBI Critical Appraisal Checklist for Case Series was used [28]. Finally, the Newcastle-Ottawa Scale was selected to evaluate the risk of bias for cohort studies [29].

Statistical Analysis

Data was collected and organised in Excel, while statistical analysis was conducted using R software (R Foundation for Statistical Computing, Vienna, Austria) to perform Linear Mixed Models (LMMs) analysis on the data set. This was to determine the statistical significance (p < 0.05) of changes in BCVA or CRT over regular intervals.

Weighted means and 95% confidence intervals (CIs) were calculated in Excel using the provided means, sample sizes, and standard deviations (SDs). In cases where SDs were not reported, the mean SD from other studies was used as an approximation. This was to avoid potential bias by allowing the inclusion of studies lacking reported SDs. Weighted means were calculated by weighing only by sample size, as the studies appeared similar in quality, and, so, this method was deemed reasonable given the available data.

To assess the statistical significance of weighted means, CIs were compared. Non-overlapping CIs were interpreted as indicating statistically significant differences, while overlapping CIs suggested that the differences were not statistically significant.

Classification of Complications

Complications were classified as either minor or major adverse effects. Major adverse effects were defined as any of the following: endophthalmitis, moderate or worse retinal tear, retinal detachment, retinal or vitreous haemorrhage, increased intraocular pressure (IOP), angle-closure glaucoma, cataract formation, moderate or worse ocular inflammation, or a moderate or greater reduction in visual acuity.

Inconsistencies in the reporting of complications across studies were anticipated [30]. If this was found to be the case, an analysis of only major adverse effects would be included to ensure consistency and reliability.

Results

The initial database search identified a total of 382 potentially related studies. Of these, 13 were removed by automated filters, and a further 93 were excluded as duplicates. The remaining 276 reports were then screened based on titles and abstracts, excluding 253 studies. The full text of the remaining 23 reports was then assessed against the inclusion and exclusion criteria. This left eight studies for inclusion, with a total of 903 eyes included. The majority of this data was provided by the PULSAR study [20].

**Figure 1:**
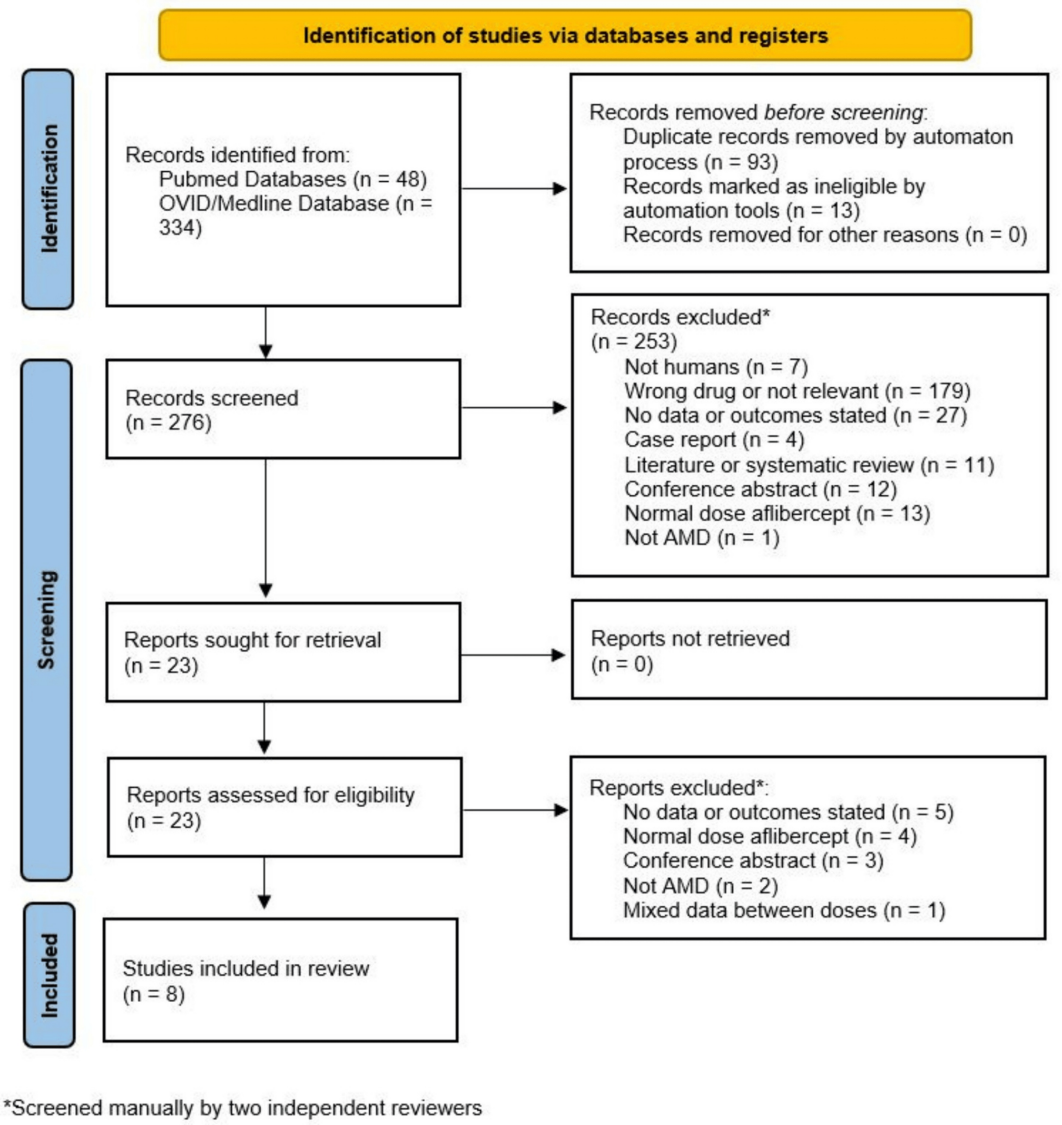
PRISMA study selection flow diagram PRISMA, Preferred Reporting Items for Systematic Reviews and Meta-analysis; AMD, Age-related macular degeneration

A summary of the basic characteristics of the included studies is presented in Table 1, and the study selection flow diagram is shown in Figure 1.

**Table 1:** Summary of baseline characteristics of included studies

Author	Year	Study design	Number of eyes	Mean age (yrs)	% female	Aflibercept dose (mg)	Patient type
Broadhead et al. [31]	2021	Retrospective cohort	9	78.7	44.4	3	Treatment resistant
Feng et al. [32]	2024	Prospective cohort	18	69.3	22.2	3	Mixed
Heier et al. [17]	2011	Randomised controlled trial	31	Not reported	Not reported	4	Mixed
Nguyen et al. [33]	2012	Randomised controlled trial	14	76.9	50	4	Mixed
You et al. [34]	2018	Retrospective case series	33	Not reported	Not reported	4	Treatment resistant
Zhang et al. [35]	2024	Retrospective cohort	73	71.3	34.3	4	Treatment resistant
Wykoff et al. [21]	2023	Randomised controlled trial	53	77	56.6	8	Treatment naïve
Lanzetta et al. [20]	2024	Randomised controlled trial	672	74.6	53.8	8	Treatment naïve

Quality of Studies

Of all four included RCTs, all were assessed to have a low risk of bias across all aspects of the Cochrane Risk of Bias 2 tool. For the included case series, most criteria from the JBI Critical Appraisal Checklist were met, but it was unclear whether there was complete inclusion of participants across a consecutive time period. Nonetheless, the overall assessment indicated a low risk of bias, so the study remained included. For the remaining three cohort studies assessed using the Newcastle-Ottawa Scale, two were scored as 6/9, and the remaining one as 5/9. For all three studies, the assessment of outcome was unclear, as it was not specified whether outcomes (BCVA and CRT) were assessed using independent blind methods, and there was no mention of record linkage. Therefore, this could have introduced potential bias into the findings in terms of data collection. However, given that CRT was consistently measured using optical coherence tomography (OCT), and there was no indication of any inconsistent reporting practices for BCVA, the likelihood of substantial bias in outcome measurement was considered low. Regardless, no points were awarded for this section in any of the cohort studies. As a result, all three were deemed of moderate quality and included in this review.

Efficacy Analysis

The primary outcomes assessed included aflibercept dose, changes in CRT, changes in BCVA, and injection frequency. To aid analysis, studies were grouped by dose into 3 mg, 4 mg, and 8 mg cohorts. Due to differences in injection frequency within the 4 mg group, this cohort was further subdivided into additional high-frequency (more injections) and low-frequency (fewer injections) groups. This classification resulted in six analysis groups: 3 mg, total 4 mg, 4 mg high-frequency, 4 mg low-frequency, 8 mg, and all studies combined.

Comparative Analysis of CRT

CRT was measured throughout the duration of all included studies. However, one study had a very short follow-up period of only one month [33]. Table 2 shows the collated CRT data from all eight studies. Not all time points were reported in every study, so missing data points were left blank. SDs were included where available.

**Table 2:** Summary of Central Retinal Thickness (CRT) outcomes across all studies

Author	Number of eyes	Dose of aflibercept (mg)	Frequency of injections (weeks)	Baseline CRT (µm) (SD)	1 month CRT (µm) (SD)	3 months CRT (µm) (SD)	6 months CRT (µm) (SD)	10 months CRT (µm) (SD)	12 months CRT (µm) (SD)
Broadhead et al. [31]	9	3	5.8	229 (35)	226 (38)	229 (37)	222 (25)	N/A	222 (36)
Feng et al. [32]	18	3	10.2	409 (56)	339 (60)	268 (42)	263 (27)	N/A	263 (23)
Heier et al. [17]	31	4	14.1	507	298	367	306	326	346
Nguyen et al. [33]	14	4	7	474 (156)	312	N/A	N/A	N/A	N/A
You et al. [34]	33	4	6.6	292 (122)	249 (111)	234 (105)	244 (103)	N/A	238 (121)
Zhang et al. [35]	73	4	14.4	376 (238)	237 (214)	253 (274)	278 (256)	337 (555)	336 (188)
Wykoff et al. [21]	53	8	7.6	517 (176)	355 (111)	339 (108)	349 (118)	357 (110)	N/A
Lanzetta et al. [20]	672	8	5.2	371 (128)	247 (60)	229 (51)	248 (79)	275 (69)	N/A

To assess the statistical significance of CRT changes over time, LMM analysis was performed for each dose group, as well as for all studies combined. For the pooled dataset, changes in CRT were statistically significant up to 10 months (p < 0.01 throughout). However, the change at 12 months was not statistically significant (p = 0.085), potentially suggesting increased variability at later time points.

Within the 3 mg group, LMM analysis revealed a statistically significant change in CRT from baseline to one month (p < 0.01). However, changes at subsequent intervals (3, 6, and 12 months) were not statistically significant.

In both the 4 mg and 8 mg groups, LMM analysis did not identify statistically significant CRT changes at any interval. As with the other findings of no significance, these should be interpreted cautiously when analysing weighted means. Subgroup sizes were small, and variability between studies may have contributed to the lack of significance.

**Figure 2:**
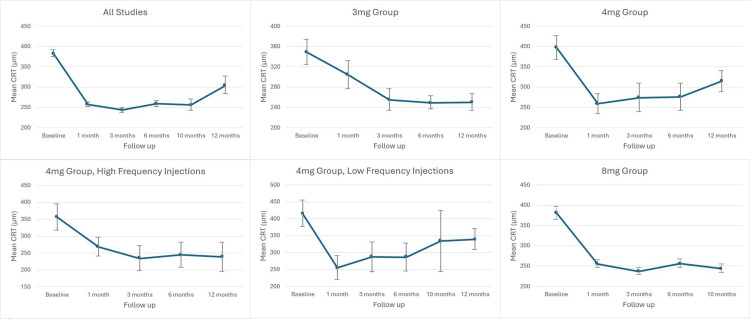
Trend in Central Retinal Thickness (CRT) over time, for the different subgroups

Weighted means and 95% CIs were calculated at each time point for each dose group. Figure 2 illustrates these CRT trends over time.

For all studies combined, CRT decreased from 382 μm at baseline to 257 μm, 243 μm, 258 μm, 256 μm, and 304 μm at 1, 3, 6, 10, and 12 months, respectively. CIs at baseline did not overlap with those at subsequent time points, indicating a statistically significant reduction in CRT of approximately 20%. However, there was an overlap between the CIs at 10 and 12 months, indicating that any change between these times was not statistically significant.

In the 3 mg group, CRT declined from 349 μm at baseline to 304 μm, 255 μm, 249 μm, and 250 μm at 1, 3, 6, and 12 months, respectively. CIs from baseline overlapped only with the one-month values, indicating statistically significant CRT reductions at 3, 6, and 12 months. This showed a 28% decrease from baseline to 12 months.

For the 4 mg group, CRT decreased from 397 μm at baseline to 259 μm, 274 μm, 276 μm, and 315 μm at 1, 3, 6, and 12 months, respectively. Baseline CIs did not overlap with those at subsequent time points, confirming statistically significant CRT reductions across all intervals, with an overall decrease of 21% from baseline to 12 months. Further analysis of the 4 mg group by injection frequency revealed the following:

High-frequency group: CRT decreased from 356 μm at baseline to 268 μm, 234 μm, 244 μm, and 238 μm at 1, 3, 6, and 10 months, respectively. While these results appeared consistent, they may lack reliability due to the limited sample size, as one study contributed much of the data.

Low-frequency group: CRT decreased from 415 μm at baseline to 255 μm, 287 μm, 286 μm, 334 μm, and 339 μm at 1, 3, 6, 10, and 12 months, respectively. CIs from baseline did not overlap with subsequent time points, indicating statistically significant reductions in CRT throughout the study period.

Finally, in the 8 mg group, CRT declined from 381 μm at baseline to 255 μm, 237 μm, 256 μm, and 244 μm at 1, 3, 6, and 10 months, respectively. Again, CIs from the baseline did not overlap with those at later time points, highlighting statistical significance. This group showed a 36% overall reduction in CRT up to 10 months.

Comparative Analysis of BCVA

Seven studies were available for analysis of BCVA, as Zhang et al.'s study did not record BCVA trends [35]. Combined with the short follow-up period of Nguyen et al.'s study, this made analysing the 4 mg subgroups extremely limited [33]. Table 3 summarises BCVA data from all studies; where data was not reported, it was left blank, and SDs are included where available.

**Table 3:** Summary of Best Corrected Visual Acuity (BCVA) outcomes across all studies

Author	Number of eyes	Dose of aflibercept (mg)	Frequency of injections (weeks)	Baseline BCVA (logMAR) (SD)	1 month BCVA (logMAR) (SD)	3 months BCVA (logMAR) (SD)	6 months BCVA (logMAR) (SD)	10 months BCVA (logMAR) (SD)	12 months BCVA (logMAR) (SD)
Broadhead et al. [31]	9	3	5.8	0.21 (0.35)	0.26 (0.36)	0.28 (0.33)	0.24 (0.34)	N/A	0.26 (0.37)
Feng et al. [32]	18	3	10.2	0.68 (0.30)	0.63 (0.29)	0.58 (0.29)	0.58 (0.3)	N/A	0.59 (0.30)
Heier et al. [17]	31	4	14.1	0.64	0.54	0.59	0.53	0.55	0.56
Nguyen et al. [33]	14	4	7	0.61 (0.27)	0.71	N/A	N/A	N/A	N/A
You et al. [34]	33	4	6.6	0.85 (0.76)	0.87 (0.92)	0.88 (0.91)	0.85 (1.39)	N/A	0.83 (0.89)
Zhang et al. [35]	73	4	14.4	0.66 (0.40)	N/A	N/A	N/A	N/A	N/A
Wykoff et al. [21]	53	8	7.6	0.54 (0.27)	0.41 (0.14)	0.40 (0.17)	0.40 (0.19)	0.38 (0.21)	N/A
Lanzetta et al. [20]	672	8	5.2	0.50 (0.25)	0.45 (0.18)	0.39 (0.20)	0.39 (0.24)	0.37 (0.23)	N/A

As with the CRT changes over time, LMM analysis was performed to assess the statistical significance of the change in BCVA over time. For the total dataset, changes in BCVA were statistically significant from baseline to 6 (p = 0.017), 10 (p < 0.01), and 12 months (p = 0.011). However, changes from baseline to one month and baseline to three months were not statistically significant.

For the 3 mg group, LMM analysis showed a statistically significant change in BCVA between baseline and one month (p < 0.01). However, no significant changes were observed at 3, 6, or 12 months. These findings align with findings from CRT analysis for the 3 mg group.

For the 4 mg group, LMM analysis found no statistically significant changes in BCVA at any interval. The change from baseline to six months was borderline (p = 0.079), suggesting a possible trend had there been more data.

Finally, in the 8 mg group, none of the BCVA changes from baseline to subsequent time points were statistically significant.

**Figure 3:**
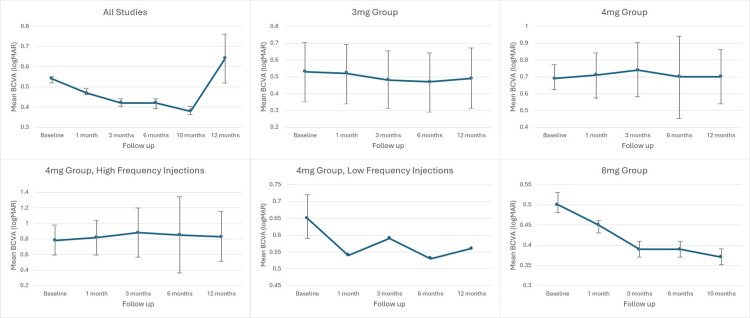
Trend in Best Corrected Visual Acuity (BCVA) over time, for the different subgroups

As with CRT analysis, the findings of non-statistical significance in LMM results should be considered carefully when evaluating weighted mean trends over time. Figure 3 shows the weighted means and CIs for each group, displaying BCVA changes over time.

For all studies combined, BCVA improved from 0.54 logMAR at baseline to 0.47 logMAR, 0.42 logMAR, 0.42 logMAR, and 0.38 logMAR at 1, 3, 6, and 10 months, respectively, before increasing again to 0.64 logMAR at 12 months. This represented a 29% improvement by 10 months. CIs for baseline and up to 10-month values did not overlap, indicating statistical significance of the mean improvement at this time point. However, the 12-month value overlapped with baseline, suggesting that the mean change at this point was not statistically significant.

In the 3 mg group, BCVA remained relatively stable, changing from 0.53 logMAR at baseline to 0.52 logMAR, 0.48 logMAR, 0.47 logMAR, and 0.49 logMAR at 1, 3, 6, and 12 months, respectively. However, CIs for these values overlapped, so the statistical significance of any changes remains uncertain.

For the 4 mg group, BCVA also remained static, from 0.69 logMAR at baseline to 0.71 logMAR, 0.74 logMAR, 0.70 logMAR, and 0.70 logMAR at 1, 3, 6, and 12 months, respectively. Further analysis of the subgroups revealed:

High-frequency group: BCVA increased slightly from 0.78 logMAR at baseline to 0.82 logMAR, 0.88 logMAR, 0.85 logMAR, and 0.83 logMAR at 1, 3, 6, and 12 months, respectively.

Low-frequency group: BCVA improved from 0.65 logMAR at baseline to 0.54 logMAR, 0.59 logMAR, 0.53 logMAR, and 0.56 logMAR at 1, 3, 6, and 12 months, respectively. However, CIs for these values could not be calculated due to a lack of SDs in the analysed study [17].

As previously mentioned, both subgroup analyses should be interpreted with caution due to the very limited sample size, with only one study contributing data.

Finally, in the 8 mg group, BCVA improved from 0.50 logMAR at baseline to 0.45 logMAR, 0.39 logMAR, 0.39 logMAR, and 0.37 logMAR at 1, 3, 6, and 10 months, respectively. CIs from baseline did not overlap with those at subsequent time points, demonstrating statistical significance. This showed a 26% improvement in BCVA up to 10 months.

Adverse Effects

Safety data were analysed across all studies. Heier et al.'s study did not provide dose-specific safety data, and so was excluded from the analysis [17]. Due to inconsistent reporting throughout the literature, data on minor complications were incomplete and could not be reliably commented on.

Among the 872 eyes included in the analysis, there were 15 recorded instances of major adverse effects. These included four cases of retinal detachment, one significant retinal tear, two retinal haemorrhages, one vitreous haemorrhage, one severe reduction in visual acuity, three instances of increased IOP, one case of angle-closure glaucoma, and two cases of cataract formation. This corresponds to a 1.7% rate of major adverse effects associated with high-dose aflibercept treatment.

Discussion

This systematic review evaluated both anatomical and functional outcomes for high-dose aflibercept treatment, in terms of changes in CRT and BCVA across the available literature.

In terms of both of these outcomes for all cases of high-dose aflibercept together, our study demonstrated a statistically significant improvement from LMM analysis and weighted means comparison when comparing baseline to results at 10 months, with an improvement of approximately 33% in CRT and 29% in BCVA.

For high-dose aflibercept as a whole, improvements in both CRT and BCVA were found at 10 months compared to baseline, with a reduction of approximately 33% and an improvement of 29%, respectively. These changes were statistically significant, and LMM analysis and weighted means comparisons also found statistical significance.

However, comparisons between different dosages or dosing intervals were limited. Most smaller dosage subgroups were not statistically significant according to LMM analyses for CRT and BCVA changes. Despite this, weighted means analysis revealed that the 8 mg group achieved larger improvements in CRT compared to the 3 mg group. Specifically, CRT improved by 36% at 10 months in the 8 mg group, compared to a 28% improvement at 12 months in the 3 mg group - both of which were statistically significant based on baseline weighted means. Additionally, the 8 mg group showed a 26% improvement in BCVA between baseline and 10 months, which was also statistically significant.

While subgroup analysis provided valuable insights into dose-specific trends, these findings must be interpreted with caution, particularly when comparing doses. The lack of statistical significance in LMM analysis for smaller groups highlights the influence of limited sample sizes. This was particularly notable in the 4 mg injection frequency subgroups, where data were often provided by only one or two studies. Consequently, it is not possible to draw true conclusions about the impact of injection frequency on outcomes or between the different subgroups.

The overall positive results align with expectations for high-dose aflibercept. The statistically significant improvements in CRT and BCVA found in this study support decisions such as the FDA’s approval of high-dose aflibercept and are consistent with findings from other studies for other indications, such as the PHOTON trial for diabetic macular oedema [19]. Additionally, the complication rate from our review aligns with those reported from other analyses of both 2 mg and 8 mg dosing, proving that high-dose aflibercept maintains a similar safety profile [12-14,19].

Despite the positive findings of this review, several limitations must be carefully considered. Firstly, analysis was limited to include only studies on nAMD. Other indications for high-dose aflibercept, such as diabetic retinopathy or retinal vein occlusion, were excluded. Consequently, the efficacy of high-dose aflibercept for these indications cannot be concluded. However, it is reasonable to assume that the safety profile would be similar. A separate and focused evaluation of high-dose aflibercept for other conditions would be necessary to provide unbiased and accurate conclusions.

Additionally, this review did not include outcomes from 2 mg aflibercept doses. This dose has been extensively studied, and its efficacy is well-established, which justified focusing on higher doses in this review [11-14]. However, this meant that a direct 2 mg comparator could not be made for these studies. Even if 2 mg outcomes had been analysed as well, it would likely have been limited, as not all studies included results for this dose.

As well as this, the search query was niche, which resulted in very few studies, restricting the availability of data for subgroup analysis. As a result, discrepancies between papers, such as differences in patient characteristics (e.g., treatment-naïve vs. treatment-resistant populations), dosing intervals, and follow-up durations, could not be thoroughly explored. This limitation introduced potential heterogeneity across the included studies. For instance, dosing frequency may influence overall treatment outcomes, and prior treatments could have affected the baseline status of the retina and subsequent changes in CRT or BCVA. Although statistical significance was achieved in some areas, these results should be interpreted cautiously, especially due to the high heterogeneity between papers.

Finally, adverse effects were not uniformly reported across all studies. This was an expected challenge and has been noted in the literature [30]. While this review supported the overall safety of high-dose aflibercept for serious adverse effects, the lack of reporting on minor complications limited a more thorough evaluation. Standardised reporting practices would allow for more accurate conclusions regarding minor adverse effects and represent an important area for improvement in future studies on anti-VEGF agents.

## Conclusions

In conclusion, this review provides important insights into the safety and efficacy of high-dose aflibercept for treating nAMD. The analysis demonstrated statistically significant improvements in both CRT and BCVA with higher doses of aflibercept, supporting its potential as an effective treatment option. Despite not having a direct comparator to outcomes from 2 mg doses, the positive findings justify the clinical application of high-dose regimens.

Additionally, this review highlights significant gaps in the current literature regarding high-dose aflibercept. Limitations in subgroup analysis, such as variations in dosing frequency and differences between dose groups, emphasise the need for further studies. Addressing these gaps will be critical for establishing optimal dosing strategies and enhancing treatment outcomes. Overall, this study adds to the growing evidence base supporting the clinical benefits of higher-dose aflibercept. However, further research is necessary to draw definitive conclusions and refine dosing protocols for nAMD.
